# Supplementary material for: The Influence of Bloom Index, Endotoxin Levels and Polyethylene Glycol Succinimidyl Glutarate Crosslinking on the Physicochemical and Biological Properties of Gelatin Biomaterials
Source: Biomolecules. 2021 Jul 9;11(7):1003. doi: 10.3390/biom11071003 (PMC8301829; doi:10.3390/biom11071003)
Supplement: Supplementary file 1 [file biomolecules-11-01003-s001.zip › biomolecules-1173295-supplementary.pdf]

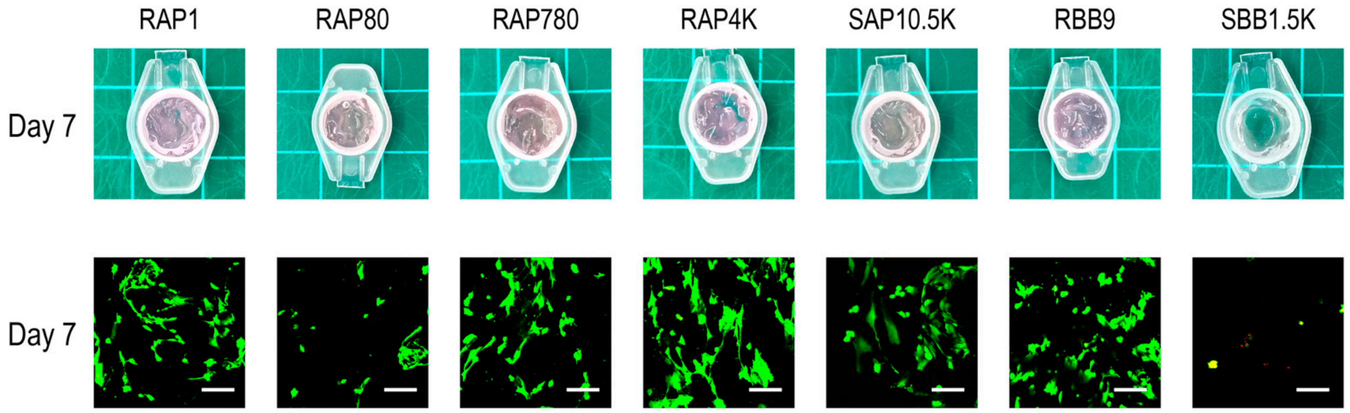

Supplementary Figure S1. 200,000 human adipose derived stem cells were seeded per 1 mM polyethylene glycol succinimidyl glutarate crosslinked gelatin hydrogel. Live cells: Green. Dead cells: Red. Scale bar: 100 µm
